# Assessment of Muscle Activity During a Deadlift Performed by Construction Workers

**DOI:** 10.3390/muscles3040029

**Published:** 2024-10-08

**Authors:** Renato Alexandre da Costa-Machado, Ana Conceição, Fernando Rocha, Marco Branco

**Affiliations:** 1 Department of Sport Sciences, Sport Sciences School of Rio Maior, 2040-413 Rio Maior, Portugal; renato.machado@esdrm.ipsantarem.pt (R.A.d.C.-M.); marcobranco@esdrm.ipsantarem.pt (M.B.); 2 Research Centre in Sports, Health and Human Development (CIDESD), 5000-801 Vila Real, Portugal; 3 Escola Superior de Educação de Setúbal, Instituto Politécnico de Setúbal, 2914-504 Setúbal, Portugal; fernandopsrocha@gmail.com; 4 Life Quality Research Centre (CIEQV), 2040-413 Rio Maior, Portugal; 5 Centre for the Study of Human Performance (CIPER), Faculdade de Motricidade Humana, Universidade de Lisboa, Cruz Quebrada-Dafundo, 1499-002 Lisboa, Portugal; 6 SPRINT Sport Physical Activity and Health Research & Innovation Center, 2040-413 Rio Maior, Portugal

**Keywords:** electromyography, deadlift, construction workers, exercise

## Abstract

Construction work is physically demanding. The tasks involved in this professional occupation physically exhaust workers and put them at high risk of injury. This work aimed to analyze muscle fatigue in civil construction workers with and without a history of physical activity. For this purpose, the muscle activity of the biceps femoris, trapezius transversalis, and erector spinae longissimus muscles was evaluated using surface electromyography. Eleven male civil construction workers aged 26 to 52 years (38.00 ± 9.60), seven of whom were untrained (N = 7) and four of whom were trained (N = 4), participated in this study. Each subject completed two assessments at two different times. A questionnaire was completed for the first assessment, and each subject’s maximum repetition for the deadlift exercise was assessed. In the second assessment, the subjects were assessed while performing the deadlift in four different situations: 50%RM at rest, 70%RM at rest, 70%RM at fatigue, and 50%RM at fatigue. The trained construction workers had higher levels of muscle activity, and there were no significant differences in muscle activation between the fatigued and non-fatigued sets. There appears to be a strong influence of physical exercise on muscle activity and fatigue in civil construction workers.

## 1. Introduction

Construction is the basis for several industries, stimulating their activities by building facilities for various purposes and contributing significantly to the economy by maintaining and increasing industrial productivity [1]. There are several risk factors for construction workers (CWs), ranging from precarious working conditions, poor or inadequate safety equipment, repetitive movements, incorrect postures, high contact forces and long hours of exposure to adverse atmospheric conditions, among others [1,2,3]. These factors contribute to a high risk of injury and death, with a twofold greater risk of injury and a threefold greater risk of death compared to other occupations [2,4,5,6,7,8,9]. According to Hulls et al. [10], the construction industry is predominantly male, male-dominated industries have a greater prevalence of risky health behaviors, and male habits may contribute to poorer health outcomes. The risk factors associated with this occupation are exacerbated by the poor health habits of CWs, such as smoking, alcohol consumption, overweight, and obesity [11,12,13,14,15]. Given their physically and psychologically demanding occupation, CWs are exposed to high levels of physical activity (PA) and subsequent daily fatigue, which promotes negative health outcomes [2,7,9,15,16]. In CWs, there are several associated health problems, such as musculoskeletal injuries, most commonly in the shoulder, knee, and lumbar regions [2,4,6,17]. Cardiovascular and metabolic issues, such as hypertension, diabetes, and cholesterol, are also observed in this population as a consequence of their lifestyle and occupation [15,18].

According to Magyari et al. [19], physical exercise (PE) and PA are recommended for all healthy adults, as well as the maintenance of PA levels at a moderate intensity for at least 150 min per week, distributed throughout the week. Although CWs have higher levels of PA, their work appears to be more detrimental than beneficial given its low intensity and long hours, highlighting the importance of structured and planned PE practices [8,20]. The tasks of this occupation appear to require high balance and postural muscle strength, with CWs carrying heavy equipment and materials on sloping floors and, in some cases, with reduced grip [2]. As these workers are exposed to several movements throughout the day, the movement of “picking up an object from the ground”, such as the deadlift (DL) exercise, which is directly transferred to the daily life of these professionals [21] and requires considerable strength and the use of postural muscles, such as the shoulder girdle and lower back, was analyzed. According to Amin et al. [22], repetitive loading of spinal tissues, especially under high-magnitude spinal loads, has been associated with a high risk of fatigue failure in spinal tissues. In addition, according to Ramirez et al. [23], performing a DL while fatiguing increases trunk flexion and is likely to place greater mechanical demands on internal trunk tissues (muscles and ligaments) to ensure spinal balance and stability, ultimately leading to greater spinal loads. Moreover, according to Martin-Fuentes et al. [24], the biceps femoris is the most studied muscle when performing the DL exercise. Still, it is the erector spinae that shows the highest activation. Ramirez et al. [23] reported that lift-off (initial part of the movement) is the lifting position associated with the greatest mechanical demand on the lumbar spine during the DL.

The main objective of this study was to verify the levels of electrical activity in the muscles of construction workers who practice physical exercise (CWPE) and construction workers who do not practice physical exercise (CWnPE), considering that greater electrical activation is an indicator of greater muscular support to the spine during the execution of movement. This study was designed to assess the hypothesis that CWPE may undergo greater electrical muscle activation and may better handle high-induced fatigue levels.

This analysis was performed to determine whether there were differences between the groups to determine whether practicing PE benefits this population. The subjects performed four sets of DL, two sets at 50%RM (repetition maximum) and two sets at 70%RM. The first two sets (50%RM and 70%RM) were performed at rest, and the last two sets (70%RM and 50%RM) were performed at fatigue.

## 2. Results

Table 1 shows that the CWPE group had greater muscle activation than the CwnPE group in every set and every muscle. The CwnPE group showed higher muscle activation in the TTL and TTR in every set, with values between approximately 20% RMS and 37% RMS, respectively. On the other hand, BFL, BFR, ESL, and ESR had values below 20% RMS in every set. The 70%RM set at post-fatigue was the set that had the greatest activation in almost every muscle for these subjects. When comparing the sets in the pre-fatigue situation, nearly all muscles reported higher muscle activation in the 70%RM set, except BFR and ESL, which did not report this behavior. In the post-fatigue situation, the untrained subjects also reported higher values in almost all muscles, except for the ESL and ESR in the 70%RM set. As in the case of CWnEP, the analysis of the CWPE group of workers showed that the muscles with higher muscular activation were the same: the TTL and TTR.

There were significant differences (*p* ≤ 0.05) in the muscle activation value of the TTL in the pre-fatigue situation between the 50%RM set (21.02% ± 12.15) and the 70%RM set (28.10% ± 9.14) (z = −2.02, *p* = 0.04) and in the muscle activation value of the TTR in the pre-fatigue situation between the 50%RM set (19.91% ± 10.13) and the 70%RM set (30.70% ± 10.26) (z = −2.02, *p* = 0.04) in the CWnPE group. In the post-fatigue situation, no significant differences were observed between the 50%RM and 70%RM sets or between either set of the CWPE.

In Table 2, when comparing the same intensity values at pre-fatigue and post-fatigue, higher values of muscle activation were observed in the post-fatigue set at both 50%RM and 70%RM. Significant differences were found between the muscle activation values in the TTL at pre-fatigue (21.02% ± 12.15) and post-fatigue (25.46% ± 18.33) (z = −2.02, *p* = 0.04) and in the TTR at pre-fatigue (19.91% ± 10.13) and post-fatigue (35.06% ± 22.86) (z = −2.02, *p* = 0.04), both at 50%RM. There were also significant differences between the TTR values in the 70%RM set when comparing the pre-fatigue and post-fatigue situations (z = −2.02, *p* = 0.04), with the TTR showing greater post-fatigue muscle activation (36.69% ± 29.61) than pre-fatigue muscle activation (30.70% ± 10.26). CWPE showed no significant differences between sets.

**Figure 1:**
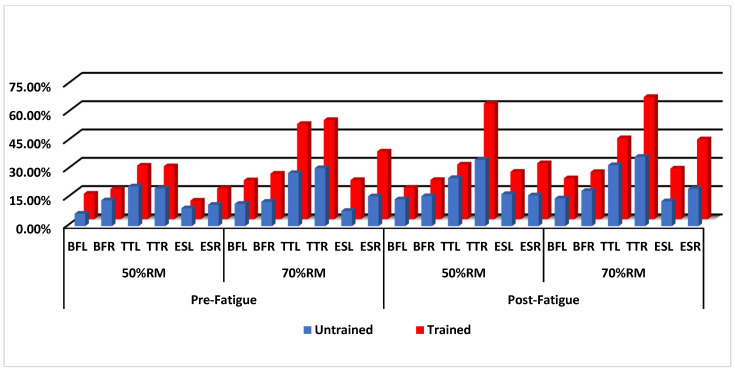
Comparison of muscle activation (RMS (%)) between 50%RM and 70%RM sets in pre-fatigue and post-fatigue situations in untrained and trained subjects. BFL: biceps femoris left; BFR: biceps femoris right; TTL: trapezius transversalis left; TTR: trapezius transversalis right; ESL: erector spinae longissimus left; ESR: erector spinae longissimus right.

Although significant differences were found between the groups, none were detected for any of the variables. Notably, the mean and standard deviation values were higher for trained subjects compared to untrained subjects (Figure 1). Muscle asymmetries (Table 3) between the left and right sides were also tested, and no significant differences were found for either group.

## 3. Discussion

This study aimed to compare the electrical activity in the muscles of two groups of construction workers: those who engage in regular physical exercise (CWPE) and those who do not (CWnPE). The underlying premise is that higher levels of electrical muscle activation are indicative of increased muscular support for the spine during movement. The primary objective was to determine whether there was a difference in the level of electrical muscle activation between the two groups. The hypothesis being tested is that CWPE may exhibit greater electrical muscle activations, suggesting that they might be better equipped to manage the high levels of fatigue induced by their work. This could highlight the importance of regular physical exercise in enhancing muscular endurance and spinal support among construction workers. Differences were observed between subjects, with the untrained subjects showing significant differences in some values of muscle activation, while the trained subjects showed no significant differences in any parameter. However, significant differences were observed only in the TTL and TTR, which may reflect the low ability of the CWnPE to maintain shoulder girdle stability.

Since CWnPE showed significant differences between sets of different intensities and with induced fatigue, the results obtained in this work seem to align with those presented in other studies [2,4,6,17]. Considering these results and considering the increased muscular difficulty of providing support for the spine during movement, it appears that the consequences of the tasks performed by CWs, caused by long working hours and high levels of PA, can lead to several health problems, such as musculoskeletal injuries.

Compared with CWnPE, CWPE showed greater muscle activation in every muscle and every set, which may reflect the influence of exercise, which seems to be consistent with the findings of Viester et al. [5], who suggested that the lack of effect produced by occupational PA may be related to the average high levels of baseline PA at work for CW, highlighting the importance of a structured and planned exercise schedule. Additionally, as van den Berge et al. [15] claim, worksite health promotion interventions should aim at reducing occupational PA, improving leisure-time vigorous PA, and reducing obesity, which would contribute to an increase in beneficial health effects.

CWnPE showed greater muscle activation in the TTL and TTR, with peak values of 32% RMS and 37% RMS, respectively, and values below 20% in the other muscles. On the other hand, CWPE showed peak values of 50% and 65% for TTL and TTR, respectively, and values above 20% activation in all muscles except BFL and BFR at pre-fatigue.

Regarding muscle asymmetries, no significant differences were found between untrained and trained subjects, and no significant differences were observed between subjects in the fatigued condition, although CWnPE presented higher mean frequency values. This could be due to the reduced size of the sample, so further investigations on this topic should include an increased sample size.

According to the results of this study, the TTL and TTR are the muscles with the greatest electrical muscle activity, followed by the ESL, ESR, BFL, and BFR. This finding is consistent with the findings of other studies [25,26] reporting that the ES has greater muscle activation than the BF; however, these findings disagree with those of Snyder et al. [27], who noted that the BF has greater muscle activation than does the ES. Nevertheless, those studies did not analyze TT muscle activity; therefore, we could not compare them. Insufficient data exist on this topic, prompting a recommendation for further exploration in future research endeavors. Given this gap, one possible reason for the higher muscle activity in the TTL and TTR could be the necessity of the CW population to control the shoulder girdle for task performance, a notion supported by Manttari et al. [2]. The tasks carried out by CWs appear to demand a significant balance between the capacity and strength of postural muscles, as they often support heavy equipment, sometimes on unstable surfaces.

Our results show a difference in the RM between CWPE and CWnPE, with CWPE showing higher values, which seems to be related to the exercise itself, with strength training being the most valid way to increase hypertrophy and muscle strength. Those higher values were also perceived for younger individuals, which is supported by Merkus et al. [7]. In this sense, the hypothesis presented is accepted, suggesting that PA can increase muscle activity and consequently provide greater muscular support to the spine during the execution of the movement, which could indicate some opportunities, such as decreasing absenteeism and preventing injuries. The introduction of formal physical exercise in the workplace is also a way to balance muscle activity in homologous muscles and, consequently, improve the ability to maintain correct postures during long working hours, which could once again result in a reduction in injuries. The results seem not entirely aligned with the findings of Stock et al. [28], who reported that subjects with low-back pain did not exhibit reductions in force or sEMG excitation. Consequently, they did not adopt a compensatory neuromuscular pattern to enhance force production. Notably, their study employed an isometric Deadlift (DL) instead of a traditional DL. The same authors also acknowledged their lack of awareness regarding previous literature on this issue, providing limited opportunities to compare those findings.

Resistance training is essential for construction workers, given the physically demanding nature of their occupation. It enhances muscular strength and endurance, which are crucial for performing tasks such as lifting, carrying, and other repetitive movements inherent in this profession. Construction workers who regularly engage in resistance training are less prone to musculoskeletal injuries. This form of training strengthens muscles and connective tissues, offering improved support for joints and reducing the risk of strains, sprains, and tears. Moreover, it contributes to overall health, aiding in weight maintenance and decreasing the likelihood of chronic conditions such as heart disease and diabetes. In addition to physical benefits, resistance training positively impacts mental health, reduces stress levels, and enhances sleep quality, resulting in improved concentration and productivity at work. Additionally, it promotes better posture and balance, serving as a preventive measure against falls and related injuries on construction sites.

## 4. Materials and Methods

### 4.1. Sample

The samples were selected by convenience and consisted of 11 male CWs (n = 11) aged 38.00 ± 9.60 years with a body mass index (BMI) of 29.43 ± 2.60 g/m^2^ (corresponding to overweight) and 15.45 ± 10.80 years in the construction sector. The CWs were divided into two distinct groups: (i) CWPE (n = 4) and (ii) CWnPE (n = 7). Exclusion criteria were defined, and participants who had any health concerns or who were on medication were excluded.

Sample characteristics were observed in terms of age, with CWnPE aged 41.14 ± 9.70 years and CWPE 32.70 ± 7.40 years; weight, with CWnPE weighing 86.07 ± 12.4 kg and CWPE weighing 88.65 ± 3.2 kg; BMI, with CWnPE having 29.26 ± 3.1 g/m^2^ and CWPE having 29.74 ± 1.7 g/m^2^; occupational time, with CWnPE presenting 18.43 ± 11.40 years and CWPE presenting 10.25 ± 8.70 years; and RM values for DL, where CWnPE presented values of 95.14 ± 19.60 kg and CWPE values of 123.00 ± 19.10 kg. Regarding the duration of PE practice, the CWPEs had between 3 and 7 years (5.00 ± 1.60) of resistance training, and the CWnPEs had no record of PE practice.

### 4.2. Procedures

**Figure 2:**
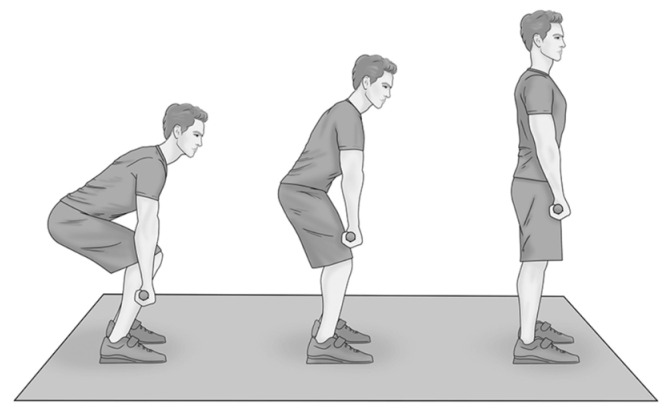
Deadlift (start and end positions).

This study received official approval from the Technical-Scientific Council of the Sport Sciences School of Rio Maior, Santarém Polytechnic University. This was a comparative study of two CW populations, CWPE and CWnPE, that is, a two-time point study. First, the subjects who volunteered to participate were informed about the nature and objectives of the study, as well as its procedure, and that they could withdraw from the study at any time, after which informed consent was signed. Participants were also informed that they could not use any type of stimulant during this trial. After receiving an introduction to the technical movement, the DL (as depicted in Figure 2), participants engaged in two sets of 10 warm-up repetitions using solely the Olympic barbell (20 kg). Following the warm-up and a detailed explanation of the exercise, participants underwent a 1RM prediction test through indirect methods. This involved completing multiple sets until the predicted 1RM value was determined. The 1RM prediction test began with a load that was expected to be applied for 8 to 12 repetitions. If the participant was able to complete 10 repetitions, a 2 min rest period was provided. A 20% increase in the previous load was then applied, and the participant performed the test again. This procedure was repeated until the participant was unable to complete 10 repetitions, at which point an estimate of the 1RM was used as described by Baechle and Groves [29]:

$$1RM=Workload(Kg)\times \left[\left(0.0375\times Reps\right)+0.978\right]$$

Second, EMG evaluation was carried out during the four sets. EMG evaluation was performed at the same time of day for every participant (5–7 pm). The first set was performed at a load of 50%RM, and the participants were asked to complete 12 repetitions. A second set was performed after a 2 min rest, this time at 70%RM, again with 12 repetitions. After the first two sets, a fatigue protocol was used in which the participants were asked to complete as many repetitions as possible at a load of 70%RM after a 2 min rest. The third set was performed at a load of 70%RM after a 2 min rest post-fatigue protocol, with 12 repetitions. The fourth set was performed at a load of 50%RM with a 2 min rest with 12 repetitions.

The purpose of the fatigue protocol was to induce physical fatigue, often experienced by CWs through exercise, and to analyze how CWs behave after reaching a high level of fatigue under various loads.

### 4.3. Instrumentation

Surface EMG signals from (i) the biceps femoris (BF), left (BFL), and right (BFR); (ii) the trapezius transversalis (TT), left (TTL), and right (TTR); and (iii) the erector spinae longissimus (ES), left (ESL) and right (ESR) muscles were measured.

Bipolar surface electrodes (10 mm diameter discs and 57 mm diameter with a 3.9 mm diameter snap connector, Plux, Lisbon, Portugal) with an interelectrode distance of 20 mm were used and placed following SENIAM recommendations [30]. The electrodes were positioned parallel to the muscle fiber orientation with an interelectrode distance of approximately 2.0 cm. The skin was prepared by shaving, abrading, and cleaning with isopropyl alcohol before electrode placement so that the interelectrode resistance did not exceed 5 Kohm [31]. The ground electrode was positioned over the seventh cervical vertebra.

### 4.4. Data Analysis

The data were collected using MonitorPlux software (PLUX Biosignals, Lisbon, Portugal), recorded in a text file (.txt), and then exported to MATLAB software (MathWorks Inc., MA, United States) for data processing. A custom MATLAB routine was prepared to (1) filter the raw EMG data with a fourth-order Butterworth digital filter at 30–300 Hz, based on the power spectrum plot, to remove noise; (2) rectify the filtered data; (3) apply a linear envelope [32]; and (4) normalize the data based on the maximum activation peak during task performance. Fatigue was determined according to Puce et al. [33] using the mean frequency (MFREQ) of the EMG data and other values, such as the absolute and relative root mean square (RMS).

For statistical analysis, Shapiro–Wilk tests of normality were run. The results showed a non-normal distribution; therefore, the Wilcoxon test was used to compare the pre- and post-fatigue tests and to compare the 50%RM and 70%RM tests. The Mann–Whitney test was used to compare the CWPE and CWnPE groups. The level of statistical significance was defined as *p* ≤ 0.05.

## 5. Limitations

This study presented several limitations, including the age gap between the participants and the reduced sample size. More studies with larger and similar samples are required to obtain more concise conclusions.

## 6. Conclusions

This article discusses muscle activation in construction workers (CWs) and its relationship with regular physical exercise. CWs who exercised regularly (CWPE) showed greater muscle activation than those who did not exercise (CWnPE), which suggests that they are better prepared to deal with high levels of work-induced fatigue. Additionally, this study highlights the importance of structured physical exercise to improve muscular endurance and spinal and lumbar support among construction workers. In conclusion, resistance training encompasses more than muscle development alone. By prioritizing health, safety, and overall well-being, construction workers can integrate it as a fundamental component of their routine.

## 7. Practical Applications

In occupational settings where lifting and manual handling are common tasks, understanding muscle activation during the deadlift can inform ergonomic interventions aimed at reducing the risk of work-related injuries. By teaching workers proper lifting techniques and providing equipment or tools that promote efficient muscle activation, employers can help prevent work-related musculoskeletal disorders.

## Figures and Tables

**Table 1:** Comparison of muscle activity (RMS (%)) between sets at 50%RM and 70%RM pre-fatigue and post-fatigue in untrained and trained subjects.

			50%RM		70%RM				
			Mean	Std Dev	Mean	Std Dev	Z	≠	Effect Size
Untrained	Pre-Fatigue	BFL	6.59	3.00	11.80	6.96	−1.83	0.07	-
		BFR	13.66	17.73	12.88	18.43	−0.37	0.72	-
		TTL	21.02	12.15	28.10	9.14	−2.02	0.04	−0.61
		TTR	19.91	10.13	30.70	10.26	−2.02	0.04	−0.61
		ESL	9.44	7.96	8.06	1.68	−0.94	0.35	-
		ESR	11.28	8.80	15.78	8.85	−0.94	0.35	-
	Post-Fatigue	BFL	14.18	8.04	14.67	10.85	−0.73	0.47	-
		BFR	15.85	19.58	18.62	25.49	−0.73	0.47	-
		TTL	25.46	18.33	32.31	25.87	−1.36	0.17	-
		TTR	35.06	22.86	36.69	29.61	0.00	1.00	-
		ESL	16.97	22.47	13.16	14.68	−0.73	0.46	-
		ESR	16.29	7.19	19.74	7.26	−0.94	0.35	-
Trained	Pre-Fatigue	BFL	13.52	10.47	20.64	20.89	−1.07	0.29	-
		BFR	16.02	5.06	24.12	6.64	−1.60	0.11	-
		TTL	28.42	9.26	50.45	24.88	−1.83	0.07	-
		TTR	28.05	28.56	52.57	45.09	−1.83	0.07	-
		ESL	9.89	1.91	20.75	15.60	−1.46	0.14	-
		ESR	16.27	5.68	35.87	24.14	−1.83	0.07	-
	Post-Fatigue	BFL	16.73	6.82	21.69	11.72	−1.83	0.07	-
		BFR	20.82	1.34	25.01	6.33	−1.07	0.29	-
		TTL	28.99	23.08	42.93	31.40	−1.10	0.27	-
		TTR	61.28	50.92	64.75	68.43	−0.37	0.72	-
		ESL	25.16	12.02	26.88	17.34	−0.37	0.72	-
		ESR	29.68	9.93	42.30	23.72	−1.46	0.14	-

RMS: root mean square; BFL: biceps femoris left; BFR: biceps femoris right; TTL: trapezius transversalis left; TTR: trapezius transversalis right; ESL: erector spinae longissimus left; ESR: erector spinae longissimus right.

**Table 2:** Comparison of muscle activity (RMS (%)) between sets pre-fatigue and post-fatigue at 50%RM and 70%RM in untrained and trained subjects.

			Pre-Fatigue		Post-Fatigue				
			Mean	Std Dev	Mean	Std Dev	Z	≠	Effect Size
Untrained	50%RM	BFL	6.59	3.00	14.18	8.04	−1.83	0.07	-
		BFR	13.66	17.73	15.85	19.58	−0.37	0.72	-
		TTL	21.02	12.15	25.46	18.33	−2.02	0.04	−0.61
		TTR	19.91	10.13	35.06	22.86	−2.02	0.04	−0.61
		ESL	9.44	7.96	16.97	22.47	−0.41	0.69	-
		ESR	11.28	8.80	16.29	7.19	−1.21	0.23	-
	70%RM	BFL	11.80	6.96	14.67	10.85	−1.10	0.27	-
		BFR	12.88	18.43	18.62	25.49	−1.10	0.27	-
		TTL	28.10	9.14	32.31	25.87	−0.14	0.89	-
		TTR	30.70	10.26	36.69	29.61	−2.02	0.04	−0.61
		ESL	8.06	1.68	13.16	14.68	−0.14	0.89	-
		ESR	15.78	8.85	19.74	7.26	−1.48	0.14	-
Trained	50%RM	BFL	13.52	10.47	16.73	6.82	−1.60	0.11	-
		BFR	16.02	5.06	20.82	1.34	−1.60	0.11	-
		TTL	28.42	9.26	28.99	23.08	−0.37	0.72	-
		TTR	28.05	28.56	61.28	50.92	−1.83	0.07	-
		ESL	9.89	1.91	25.16	12.02	−1.46	0.14	-
		ESR	16.27	5.68	29.68	9.93	−1.83	0.07	-
	70%RM	BFL	20.64	20.89	21.69	11.72	0.00	1.00	-
		BFR	24.12	6.64	25.01	6.33	−1.07	0.29	-
		TTL	50.45	24.88	42.93	31.40	−0.37	0.72	-
		TTR	52.57	45.09	64.75	68.43	−0.73	0.47	-
		ESL	20.75	15.60	26.88	17.34	−1.83	0.07	-
		ESR	35.87	24.14	42.30	23.72	−1.83	0.07	-

RMS: root mean square; BFL: biceps femoris left; BFR: biceps femoris right; TTL: trapezius transversalis left; TTR: trapezius transversalis right; ESL: erector spinae longissimus left; ESR: erector spinae longissimus right.

**Table 3:** Comparison of muscle activity (mean frequency) between untrained and trained subjects in the post-fatigue situation in the 50%RM and 70%RM sets.

		Trained		Untrained			
		Mean	Std Dev	Mean	Std Dev	Z	≠
50%RM	BFL	48.16	45.59	88.85	12.23	−1.51	0.13
	BFR	42.55	48.10	47.46	32.15	−0.19	0.85
	TTL	56.28	28.17	72.85	6.15	−0.94	0.34
	TTR	67.89	14.26	58.19	5.67	−1.51	0.13
	ESL	63.83	29.80	72.69	19.79	−0.19	0.85
	ESR	58.33	12.46	60.29	6.38	−0.38	0.71
70%RM	BFL	48.10	45.58	84.10	11.65	−1.13	0.26
	BFR	40.85	52.41	50.07	34.10	0.00	1.00
	TTL	59.00	28.33	68.68	5.50	0.00	1.00
	TTR	69.43	16.56	62.40	8.19	−0.76	0.45
	ESL	62.95	29.99	74.27	21.73	−0.57	0.57
	ESR	61.05	13.10	57.70	7.70	−0.19	0.85

BFL: biceps femoris left; BFR: biceps femoris right; TTL: trapezius transversalis left; TTR: trapezius transversalis right; ESL: erector spinae longissimus left; ESR: erector spinae longissimus right.

## Data Availability

The data presented in this study are available on request from the corresponding author.
